# Assessing perceptions about critical thinking, motivation learning strategies in online psychiatric and mental health nursing education among Egyptian and Saudi undergraduate nursing students

**DOI:** 10.1186/s12912-023-01264-2

**Published:** 2023-04-10

**Authors:** Nora Ghalib AlOtaibi, Amira Alshowkan, Neama Kamel, Ayman Mohamed El-Ashry, Nagla Saleh AlSaleh, Eman Sameh Abd Elhay

**Affiliations:** 1 Department of Community Nursing, College of Nursing, Imam Abdulrahman Bin Faisal University, Dammam, Saudi Arabia; 2 Lecturer of Psychiatric and Mental Health Nursing, Faculty of nursing, Mansoura University, Mansoura, Egypt; 3 Lecturer of Psychiatric and Mental Health Nursing, Faculty of nursing, Alexandria University, Alexandria, Egypt

**Keywords:** Critical thinking, Motivation learning strategies, Online psychiatric, Mental health nursing, Education.

## Abstract

**Background:**

The pandemic context prompts nursing students to be involved in online learning. Researchers indicated that critical thinking develops through the learning process, but the link between critical thinking and online learning in nursing does not have sufficient evidence.

**Aim of Study:**

This research examines student perceptions about critical thinking, motivation, and learning strategies in online psychiatric nursing education among nursing students at Imam Abdulrahman bin Faisal University in Saudi Arabia and Alexandria University in Egypt.

**Subject and Methods:**

An online survey was designed to collect data anonymously. A total of 75 Saudi undergraduates and 105 Egyptian nursing students who met the inclusion criteria participated. The online survey assessed three parts: socio-demographic data, critical thinking motivational, and cognitive processing strategy scales.

**Results:**

The current study found that using a motivating method for online learning inspired students to engage in critical thinking and cognitive processing strategies in a psychiatric and mental health nursing course, even in two different contexts.

**Conclusion:**

This study demonstrates that using motivational methods for online learning encourages students to engage in critical thinking and cognitive processing strategies in psychiatry and mental health care courses, even in two different settings.

**Supplementary Information:**

The online version contains supplementary material available at 10.1186/s12912-023-01264-2.

## Background

COVID-19 has a global impact on education; learning has been disrupted in several institutions, most notably nursing colleges in Saudi Arabia and Egypt. Conventional in-person classes had to be suspended to maintain the welfare of all students, teaching faculty, and patients [1, 2]. In response to the pandemic, more than 170 countries now rely on online learning [3]. As a result, the psychiatric nursing course has been taught virtually at Imam Abdulrahman and Alexandria University. In psychiatric nursing curricula, nurses must be able to think critically to carry out complex nursing actions that affect patient safety and satisfaction [4].

The pandemic context prompts nursing students to address different approaches to learning strategies used by educational institutions to take full advantage of the learning experience by getting students involved in this new virtual learning setting [5]. Critical thinking is “the act of formulating a reflective judgment about what to believe or what is the priority action based on specific situations” [6]. It’s crucial to be mastered by nursing students [7, 8].

In the literature, critical thinking (CT) can be traced back to 1965 in nursing education, but it became more prominent in the 1980s as researchers endeavored to understand CT in nursing education. The use of CT during online nursing education was closely investigated in the 1990s as a result of the development of online modes, such as electronic learning systems and online audiovisual programs, which allowed students and teachers to interact as if they were in a real-world setting [9, 10]. CT is the method of self-regulatory decision-making with intent [11]. Interactive interpretation, presumption, analysis, and assessment are rationally regarded as part of the logical thinking process [11]. People are directed to make centering and informed decisions based on their beliefs using this approach [11, 12]. The foundation of professional judgment is known as CT [13]. For nurses to correctly assess nursing diagnoses, CT skills are necessary because accurate analysis of patient data can be difficult. After all, high levels of thinking and intellect are needed; the thinking skills of nurses differ and can be enhanced, and the best use of CT abilities encourages the validity of the diagnosis based on the evidence used in research [14]. Several studies reported the importance of the use of critical thinking in psychiatric and mental health nursing [15, 16]. For example, the use of well-designed simulations in psychiatric and mental health nursing education was found to enhance students’ critical thinking, decision-making, and communication skills [17]. Therefore, this study will enhance the previous studies regarding the importance of the use of critical thinking during online education.

Online learning refers to using cyberspace to reach educational resources and interact with the content, teachers, and other pupils. It helps students and instructors interact during the learning session to obtain information and build personal meaning [18]. Nurse educators play a vital role in identifying and implementing learning modifications in acquiring CT, besides focusing on innovative methods of teaching [19]. Since 2013, CT research focusing on the use of online education for psychiatric nursing has been infrequent and vague, as some studies focus on online education while others use blended methods with little evidence of their effectiveness [20, 21]. Even though there have been several systematic reviews on CT in the field of nursing studies, only a tiny number have focused on the concepts and approaches for instructors to improve online technology utilization [19]. Furthermore, it has been reported that there needs to be more research on the predictors of CT, not only in online nursing education but also in nursing education in general [22].

The academic motivation was identified as an inherent and persistent desire of the learner to investigate new concepts [23]. There are three domains of academic motivation which are intrinsic and extrinsic motivation and lack of motivation which has been proven related to self-learning of students [24–26]. In nursing students, academic motivation has been studied and showed that motivational strategies are the most important elements that enhance student self-learning skills [27, 28]. However, no published research was found to focus on the motivation strategies in psychiatric and mental health nursing during online teaching, so, this research will help to fill in the gap in this regard.

Strategies of online learning in form of self-directed learning skills and motivational approaches were used widely and proved to be effective. The use of self-directed strategies was established to enhance students learning autonomy to meet any changes in learning setting [29]. This strategy of learning enhances students’ thinking ability and self-management which was used during online education [30, 31]. For instance, during the COVID-19 epidemic, self-directed learning, and motivational learning strategies among 1187 nursing students from Saudi Arabia, the Philippines, and Thailand were examined and found that the predictors of nursing students’ self-learning were the intrinsic motivation [32].

Critical thinking is essential in clinical nursing and a core part of an online nursing education program because it can help students learn more than traditional classroom teaching can [33, 34]. According to Salyers et al. (2014), students are looking for ways to better incorporate technology into education as they become more technologically savvy [35]. According to Guri-Rosenblit, (2014), several online technologies used to aid the development of higher-order thinking skills in nursing education are used primarily to provide lectures and discussion groups rather than as the primary educational medium that would enhance CT [36]. It is worth noting that student’s perception of constructivist learning environments was related to critical thinking self-management strategies [37].

Researchers indicated that CT develops through the learning process, but the link between CT and online learning in nursing does not have sufficient evidence [33, 38]. While the literature agreed that CT could occur in an online environment, more current research on the outcomes of online learning as they relate to the CT process is needed, as this has implications for its future use in nursing education [20, 21]. However, it has been reported that the traditional educational approach impedes the development of CT skills, such as classroom teaching and the instructor-student power dynamic [19]. On the other hand, Gould et al. (2014) recommended that nursing online education and learning have definitely improved students’ learning and ability to gain CT and nursing competency, in addition to improving student satisfaction concerning accessibility and the capability to work remotely [39]. Lahti et al. (2014) also revealed that e-learning might help students gain knowledge and skills more quickly than face-to-face, teacher-centered methods [40].

The flexibility of online education for students and instructors has been studied. The positive aspect of learning has been reported as it provides flexibility in the use of different educational strategies, time, duration, and space conveniences, quick access for both instructors and students, and enhances the profound learning experience [35, 41, 42]. In addition, online learning stimulates student-centered learning, as the student manages online courses efficiently, which improves knowledge gain; self-efficacy, enhances performance, fosters CT, and enhances writing skills for the majority of students [43, 44].

As a result of the coronavirus pandemic (COVID-19), governments released decrees on quarantine, social distancing, and staying at home; therefore, colleges and universities worldwide were closed [45]. COVID-19 has imposed education schemes worldwide to use alternatives for teaching and learning [46]. As a result, during the extraordinary COVID-19 outbreak, online distance learning programs were the only available mode of education [47–49]. Therefore, IAU, Alexandria University, and its College of Nursing have been involved in developing dynamic and contemporary online clinical curricula by applying CT and solving clinical problems.

In Saudi Arabia and Egypt, online modalities in clinical and mental health nursing are still relatively new. Since then, no research has been done on the usefulness of learning approaches for improving CT abilities that aid student’ professional problem-solving abilities [50, 51]. As a result, this study aims to evaluate students’ attitudes toward critical thinking, motivation, and learning strategies in online psychiatric and mental health nursing education at Imam Abdulrahman Bin Faisal University in Saudi Arabia and Alexandria University in Egypt.

### Study design and setting

A comparative cross-sectional research design was conducted among nursing students registered to take the Psychiatric Nursing and Mental Health course between Imam Abdulrahman Bin Faisal University (IAU) in Saudi Arabia and Alexandria University in Egypt. All nursing students were registered in the Psychiatric Nursing and Mental Health course for 2021–2022. The Psychiatric Nursing and Mental Health course was taught through 6 credit hours (3 h theatrical and 3 h clinical) in both universities. Both universities taught the same course description based on the same National Academic Reference Standard (NARS), with the primary focus being the integration of knowledge and skills to provide high-quality nursing management to clients with emotional and psychiatric problems in various healthcare settings. Clients with various mental disorders and their families will receive high-quality, secure home health care. Emphasis will be placed on the fundamental understanding of the mental illness concept, mental disorder classification, clinical illustrations, critical thinking, and treatment options. The course covers ideas about the nursing process, which serve as a framework for the psychiatric nursing care plan. Additionally, the course aims to assist students in promoting mental health, recognizing and preventing mental illness, and participating in mental health restoration [52, 53].

### Participants: sample size calculation and sampling technique

Based on data from literature **Charan, & Biswas, )2013)** [54], considering level of significance of 5%, and power of study of 80%, and based on data from literature, the sample size can be calculated using the following formula:

$${\text{n}} = \frac{{({\text{Z}}1 - \alpha /2)\widehat {}2.{\text{SD}}\widehat {}2}}{{{\text{d}}\widehat {}2}}$$

Where, Z_1-α/2_ = is the standard normal variate, at 5% type 1 error it is 1.96, SD = standard deviation of variable and d = absolute error or precision. So,

$${\text{n}} = \frac{{(1.96)\widehat {}2.(0.042)\widehat {}2}}{{\left( {0.00615} \right)\widehat {}2}} = 179.2$$

Based on the above formula, the sample size required for the study is 180.

**Fig. 1:**
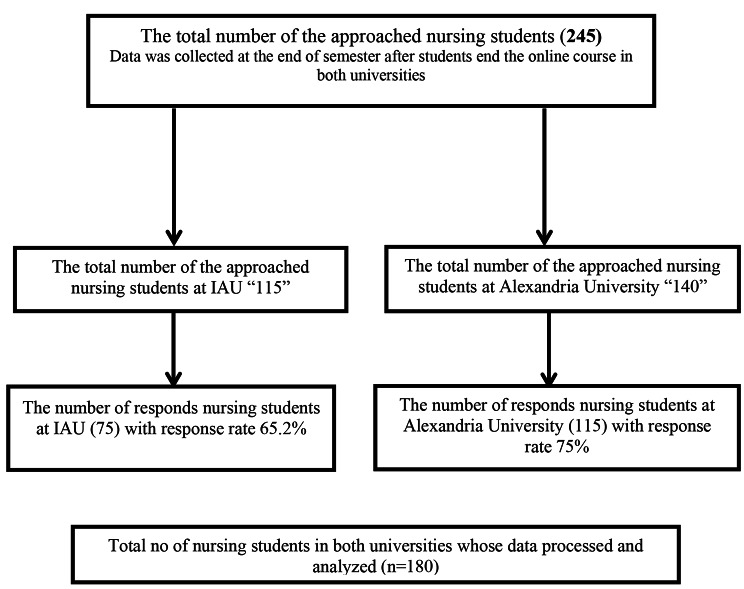
Participants’ Recruitment Process Flow Chart

All participants were chosen conveniently. A total of 75 Saudi undergraduate students responded out of 115 with a response rate of 65.2%, and 105 Egyptian undergraduate students responded out of 140 with a response rate of 75% (Fig. 1**)**. Finally, 180 nursing students representing two distinct countries and universities (IAU) in Saudi Arabia and Alexandria University in Egypt produced sufficiently detailed responses to be included in the study. The Psychiatric and Mental Health Nursing course was taught in English. The following criteria were used to determine the inclusion of the studied subject: Enrolled in psychiatric and mental health nursing at both IAU and Alexandria Universities in the second semester of the academic year 2021–2022 and willing to be involved in the study.

### Data collection

The researchers used a three-section-based questionnaire for data collection. Part 1 includes the socio-demographic data which includes the following questions: gender, marital status, age, university, which nursing course the student is studying, current GPA, hours of study, and if the student was transferred from another college or university (**supplementary file**).

## Part 2 includes Critical thinking motivation scale and The Cognitive Processing Strategies Scale

### Critical thinking motivation scale (CTMS) (English version)

It was developed by Valenzuela et al., 2011. The scale is based on a theoretical option that gives the perspective of motives primacy over the perspective of attitudes, with the aim of acquiring insight into students’ motivation levels and determining the specific component that influences their performance. [55]. CTMS measures the different components of motivation with respect to critical thinking. This scale contains 19 items on a 6-point Likert scale that allow participants to express their level of agreement or disagreement with a set of statements relating to each participant’s expectations of thinking in a critical or rigorous manner (expectation) and the importance (value) of thinking in this manner. The rest of the statements are about the value and utility (utility) they notice in thinking, the cost they are willing to take for thinking in that way thoroughly, and the interest this method of thinking arouses in them. The reliability for the scale is 0.77, and the validity has shown a significant correlation (r = 0.50–0.57) between motivation and critical thinking [55]. The Cronbach’s alpha reliability estimate in the present study was 0.912.

### The cognitive Processing Strategies Scale (CPSS) (English version)

**It** was a modified version of the Motivated Strategies for Learning Questionnaire (MSLQ) of Pintrich’s instrument. Pintrich et al. (1991) proposed five sub-scales (i.e., rehearsal (REH), elaboration (ELA), organization (ORG), critical thinking (CT), and metacognitive self-regulation (MSR); it had 31 items, each item on a seven-point Likert scale [56]. Compared with the original MSLQ, the 18-item Cognitive Processing Strategies Scale modified by Professor Jiling Liu in 2019 has a different aim: to improve equivalence and efficiency in measuring cognitive and motivational learning processes by using more cost-effective analytical procedures and minimizing the number of tests taken. The scale has 18 items on a 7-point Likert-type scale with scores ranging from 1 to 7, and the statement “When I study for this class, I practice saying the materials to myself over and over” has a score of 1 for not at all true of me and a score of 7 for very true of me. The modified scale of the Cognitive Processing Strategies Scale (CPSS) proposed five sub-scales like the original one: rehearsal (REH), represented by 2 items; elaboration (ELA), represented by 7 items; organization (ORG), represented by 2 items; critical thinking (CT), represented by 6 items; and general cognitive strategies (GCS), represented by 13 items. A lot of items had bi-factor and tri-factor categories in the subscales. The scale’s validity is 0.892, and the reliability is 0.825 [57]. In addition, Cronbach’s alpha reliability estimate in the present study was 0.892.

## Methods

### Ethical approval

This study was approved by institution review board of Imam Abdulrahman Bin Faisal University (IAU) research Ethical Committee for the study settings to gather the necessary data (11/2022). The study’s goal was communicated to the participating nursing students through their official emails. Every student was informed that they had the right to decline participation or quit at any time before finishing the study materials without repercussions. Nursing students who agreed to participate in the study provided electronic, signed, informed consent. The concept of anonymity was honored. During the study, data privacy was guaranteed.

### Data collection phase

Official permissions from the faculty of nursing IAU and Alexandria University were obtained to conduct the study. A list of the official emails of all enrolled nursing students at the psychiatric and mental health nursing course from the registration desk was obtained. The questionnaire link was sent individually to every student official university emails. It was worse mentioned that the email of nursing students was linked with national identifier.The nursing students who clicked the link were directed to the Microsoft Office Forms. To minimize the missing data, the participants were requested to fill in all items in the online questionnaire or else they could not proceed to the next page; a notification box indicated a warning note that one or more items were not answered. On completion of the questionnaire, the participant was directed to click the submit option, and finally, the online questionnaire was sent to the drive.

A pilot study was conducted to evaluate the applicability, clarity, and usefulness of the study tools. The test was administered to 30 nursing students who were not research participants. The results of the pilot showed that no modifications were required. The Alpha Cronbach’s test, which was used to evaluate the internal consistency of the research instruments, indicated that the study tools had excellent reliability. Moreover, the information was gathered at the end of semester 2021/2022.

Psychiatric and mental health nursing courses are offered in an online education mode even after the era of COVID-19.The online education modality was formed in both universities on the Blackboard platform, Zoom, and Microsoft Teams, where academic materials and assignments were posted and class activities could be completed. Reference, guidance, and effective communication were available to students. The online course content was given weekly and had the same objectives and outline as the traditional face-to-face content, but the delivery mode was different. Before starting the course, the course coordinators prepared the time plan, the schedule of theory lectures, and all teaching materials and posted them on the blackboard. First, each rotation’s objectives, time plan, and rubrics were posted on the board as a pdf file to give the students holistic information about the rotation. Secondly, the theory lecture schedule list was posted to the platform as a PDF document. Finally, all lectures were uploaded as PowerPoint files, with assignments for each lecture, videos, and references.

### Statistical analysis

Comparing the socio-demographic features of the students at IAU University and Alexandria University necessitated the analysis of demographic data using the percent (%) and chi test. The statistical analysis was conducted using SPSS V.24 (IBM Corp., Armonk, NY). To accurately describe the data, descriptive statistics revealed that the average values of continuous variables produced the correct results. Furthermore, the Critical Thinking Motivational Subdomains Scale was compared using the mean and standard deviation. The correlation coefficient was employed to compare the critical thinking motivating scale with the student assessments of the cognitive processing techniques scale. Moreover, the T- and ANOVA tests were utilized to demonstrate the connection between the students’ socio-demographic characteristics and the Critical Thinking Motivational Domains subscale. The dependability of the scale was assessed using an internal consistency and reliability test.

## Results

Table 1 shows the comparison of the socio-demographic characteristics of the students at IAU and Alexandria University: Of the 180 students surveyed, 75 were from IAU and 105 were from Alexandria University. The age range of the participants was 20 to 23 years. (69.3% and 81.0%) of IAU and Alexandria students were female, respectively. The majority of them (85.3 and 90.5%) were not married. As for GPA, (78.7%) obtained a 3.6–4.5 from IAU students, while (87.6%) of students obtained a 3.6 or less from Alexandria University. Approximately half (50.7% and 53.3%) of students reported studying 3–4 h a day. And most of them have not transferred from another university or college to the faculty of nursing. In terms of age and GPA, there is a significant difference between students at IAU University and Alexandria University (p = < 0.001**).

**Table 1:** Comparison of the socio-demographic characteristics of the students

	IAU University (n = 75)		Alexandria University (n = 105)		Chi-Square	
	n	%	n	%	X^2^	P
Gender						
Male	23	30.7	20	19.0	3.248	0.071
Female	52	69.3	85	81.0		
Marital Status						
Not Married	64	85.3	95	90.5	1.123	0.289
Married	11	14.7	10	9.5		
Age (Years)						
20 < 21	22	29.3	10	9.5	61.855	< 0.001**
21 < 22	42	56.0	20	19.0		
22 < 23	8	10.7	17	16.2		
> 23	3	4.0	58	55.2		
GPA^a^						
< 3.6	5	6.7	93	88.5	115.381	< 0.001**
3.6–4.5	59	78.7	12	11.5		
> 4.5	11	14.7	0	0		
On average, how many hours per day do you spend studying (Hours)						
≤ 1	11	14.7	16	15.2	2.310	0.511
3–4	38	**50.7**	56	**53.3**		
5–6	17	22.7	27	25.7		
≥ 7	9	12.0	6	5.7		
Did you transfer from another university or college into the nursing?						
No	62	82.7	94	89.5	1.780	0.182
Yes	13	17.3	11	10.5		

Chi square test p significant at < 0.001** IAU: Imam Abdulrahman bin Faisal University

^a^ GBA’s total score was five at IAU University and four at Alexandria University

### Test of normality

Table 2 shows the normality test of the critical thinking motivational scale and student perception of the cognitive processing strategies scale. The table revealed that the test of normality by Shapiro-Wilk value was insignificant, which reflects that it is reasonable to behave as if the data set is (sufficiently close to being) normally distributed.

**Table 2:** Normality test of Critical thinking motivational scale and student perception of Cognitive processing strategies scale

	IAU University (n = 75)		Alexandria University (n = 105)	
	Shapiro-Wilk Value	P – Value	Shapiro-Wilk Value	P – Value
Critical thinking motivational scale	0.964	0.785	0.967	0.754
Cognitive processing strategies scale	0.987	0.436	0.983	0.391

P value at ≤ 0.05

### Comparison of the critical thinking motivational scale domains

Table 3 compares the averages of the Critical Thinking Motivational Scale domains. According to the data in Table 2, the mean IAU score in the expectancy and cost domains is (4.2) and (4.6), respectively, higher than the mean score of Alexandria University (4.0) and (4.5). However, these differences don’t reach a statistically significant level. It also appears from the table’s data that the mean IAU score according to attainment domain, utility domain, and interest domain (5.1), (5.2), and (5.1) is less than the mean score among Alexandria University (5.2), (5.4), and (5.2). However, these differences don’t reach a statistically significant level.

**Table 3:** Comparison of Critical Thinking Motivational Scale Domains Average

	IAU University (n = 75)	Alexandria University (n = 105)	T-Test	
	Mean ± SD	Mean ± SD	t	P
1-Expectancy Domain	4.2 ± 1.2	4.0 ± 0.9	1.427	0.155
2-Attainment Domain	5.1 ± 1.2	5.2 ± 0.9	1.017	0.310
3-Utility Domain	5.2 ± 1.2	5.4 ± 0.9	1.201	0.232
4-Interest Domain	5.1 ± 1.2	5.2 ± 0.8	0.626	0.532
5-Cost Domain	4.6 ± 1.2	4.5 ± 1.1	0.678	0.499
Total Score of CTMS	10.4 ± 3.1	10.1 ± 2.3	0.535	0.594

IAU: Imam Abdulrahman bin Faisal University p value at ≤ 0.05

### Comparison of the student perceptions about cognitive processing strategies

The results of the T-test from Table 4 show the number and distribution of student perceptions about cognitive processing strategies between IAU and Alexandria University. In the IAU, the mean total score of items was (92.1 ± 22.5). It is less than the mean total score of items at Alexandria University (94.1 ± 18.5), and the result of the t-test (0.636) came with a probability value of (0.526) more significant than the significance level (0.05), and accordingly, there are no significant differences.

**Table 4:** Number and distribution of Student perceptions about Cognitive processing strategies Scale

	IAU University (n = 75)	Alexandria University (n = 105)	T-Test	
	Mean ± SD	Mean ± SD	t	P
1. When I study for this class, I practice saying the materials to myself over and over. ^CT, GCS, and REH^	5.3 ± 1.5	5.2 ± 1.4	0.406	0.685
2. I make lists of important items for this this class and memorize the lists. ^CT and GCS, REH^	5.1 ± 1.7	5.4 ± 1.5	1.534	0.127
3. When I study for this class, I pull together information from different sources such as lectures, readings, and discussions. ^ELA and GCS^	5.2 ± 1.7	5.4 ± 1.5	0.819	0.414
4. When studying for this class, I try to relate the materials to what I already know. ^ELA and GCS^	5.7 ± 1.4	5.7 ± 1.4	0.144	0.886
5. When I study for this class, I write brief summaries of the main ideas from the materials and my class notes. ^ELA and GCS^	5.0 ± 1.9	5.4 ± 1.6	1.459	0.146
6. I try to understand the materials in this class by making connections between the readings and the concepts from the lectures. ^ELA and GCS^	5.3 ± 1.6	5.6 ± 1.4	1.436	0.153
7. I try to apply ideas from other class activities such as lectures and discussions. ^ELA and GCS^	5.3 ± 1.6	5.4 ± 1.5	0.453	0.651
8. I make simple charts, diagrams, or tables to help me organize class materials. ^ELA and ORG^	4.9 ± 2.0	5.2 ± 1.5	1.143	0.254
9. When I study for this class, I go over my class notes and make an outline of important concepts. ^CT, GCS, and ORG^	5.2 ± 1.7	5.4 ± 1.5	0.635	0.526
10. I often find myself questioning things I hear or read in this class to decide if I find them convincing. ^CT and GCS^	5.4 ± 1.5	5.2 ± 1.5	0.897	0.371
11. When a theory, interpretation, or conclusion is presented in class or in the readings, I try to decide if there is good supporting evidence. ^CT and GCS^	5.2 ± 1.7	5.2 ± 1.4	0.057	0.954
12. I treat the class materials as a starting point and try to develop my own ideas about it. ^CT and GCS^	5.0 ± 1.6	5.2 ± 1.4	0.859	0.391
13. Whenever I read or hear an assertion or a conclusion in this class, I think about possible alternatives. ^CT and GCS^	4.9 ± 1.7	5.0 ± 1.3	0.690	0.491
14. When studying for this class, I make up questions to help focus on learning materials. ^ELA and GCS^	5.0 ± 1.8	5.2 ± 1.4	0.970	0.333
15. When I become confused about something I’m studying for this class, I go back and try to figure it out. ^ELA and GCS^	5.6 ± 1.6	5.2 ± 1.4	1.668	0.097
16. If the class materials are difficult to understand, I change the way I study the materials. ^GCS^	5.2 ± 1.8	5.3 ± 1.4	0.766	0.445
17. When I study for this class, I set goals for myself in order to direct my activities in each study period. ^GCS^	3.8 ± 1.2	3.9 ± 1.1	0.630	0.530
18. If I get confused taking notes in class, I make sure I sort it out afterwards. ^GCS^	5.1 ± 1.7	5.1 ± 1.6	0.205	0.838
Total Score	**92.1 ± 22.5**	**94.1 ± 18.5**	**0.636**	**0.526**

Independent sample t-test p value at ≤ 0.05 IAU: Imam Abdulrahman bin Faisal University

CT: critical thinking GCS: general cognitive strategies REH: rehearsal ELA: elaboration; ORG: organization

### Correlations among critical thinking motivational scale score and the student perception of cognitive processing strategies, Imam Abdulrahman bin Faisal University

**Fig. 2:**
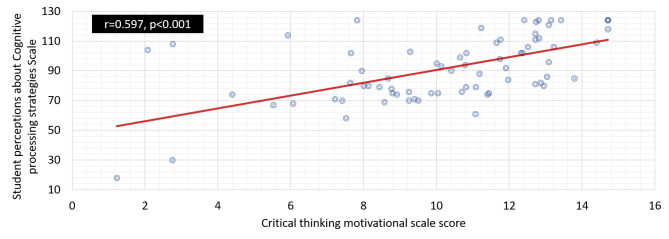
Correlation between Critical thinking motivational scale and student perceptions about Cognitive processing strategies faculty of nursing, Imam Abdulrahman bin Faisal University

As shown in Fig. 2, the Critical Thinking Motivational Scale Score is significantly correlated with the student perception of cognitive processing strategies as measured by the correlation coefficient (0.597) at the significance level of < 0.001. This correlation suggests that students with higher levels of critical thinking motivation are more likely to perceive that they use cognitive processing strategies when studying.

### Correlations among critical thinking motivational scale score and the student perception of cognitive processing strategies, Alexandria University

**Fig. 3:**
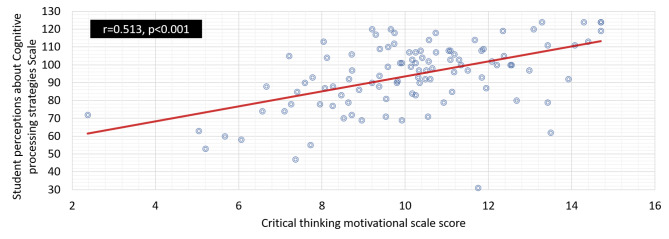
Correlation between Critical thinking motivational scale and student perceptions about Cognitive processing strategies faculty of nursing, Alexandria University

It is clear from Fig. 3 that there is a positive correlation between the critical thinking motivational scale and student perceptions of cognitive processing strategies, scaling the Alexandria University correlation coefficient function value (0.513) to a significance level of < 0.001. This suggests that when students perceive themselves to be more motivated to engage in critical thinking, they are more likely to use effective cognitive processing strategies.

### Relationships between socio-demographic characteristics of the students and student perceptions about cognitive processing strategies Scale, IAU and Alexandria University

T-Test and ANOVA results from Table 5 revealed an association between IAU students that how many hours a day do you spend studying and Critical Thinking Motivational Scale Domains Average (6.027, p = < 0.001). Specifically, the results showed that IAU students who are spent more hours studying, they had a higher average on the Critical Thinking Motivational Scale Domains.

**Table 5:** Association of the socio-demographic characteristics of the students and Critical Thinking Motivational Scale Domains Average

	IAU University Score of motivation	Alexandria University score of motivation
	Mean ± SD	Mean ± SD
Gender		
Male	10.6 ± 3.3	10.8 ± 3.2
Female	10.3 ± 3.1	10.0 ± 1.9
T [p]	0.430 [0.669]	1.442 [0.152]
Marital Status		
Not Married	10.5 ± 3.0	10.1 ± 2.63
Married	9.3 ± 3.7	10.5 ± 2.2
T [p]	1.218 [0.227]	0.461 [0.646]
Age (Years)		
20 < 21	9.9 ± 3.0	10.9 ± 2.2
21 < 22	10.7 ± 2.7	10.5 ± 2.2
22 < 23	9.0 ± 5.2	10.2 ± 2.1
> 23	12.7 ± 0.3	9.9 ± 2.4
F [p]	1.406 [0.248]	0.817 [0.488]
GPA		
< 3.6	10.3 ± 1.7	10.1 ± 2.3
3.6–4.5	10.2 ± 3.2	10.1 ± 1.9
> 4.5	11.3 ± 3.4	10.7 ± 1.3
F [p]	0.593 [0.555]	0.070 [0.933]
On average, how many hours per day do you spend studying (Hours)		
≤ 1	9.9 ± 3.8	9.6 ± 1.7
3–4	10.9 ± 2.7	10.3 ± 2.3
5–6	11.3 ± 2.1	10.1 ± 2.7
≥ 7	6.8 ± 3.4	10.5 ± 1.2
F [p]	6.027 [< 0.001]	0.385 [0.764]
Did you transfer from another university or college into the nursing?		
No	10.4 ± 3.2	10.2 ± 2.1
Yes	10.2 ± 3.0	9.5 ± 3.2
T [p]	0.150 [0.881]	1.026 [0.307]

IAU: Imam Abdulrahman bin Faisal University one way ANOVA test p value < 0.001

### Relationships between socio-demographic characteristics of the students and critical thinking motivational scale domains average, IAU and Alexandria University

T-test and ANOVA result from Table 6 revealed an association between the Alexandria University students transferring from another university or college into the nursing program and their perceptions of the Cognitive Processing Strategies Scale (2.130, p = 0.036). These results indicate that students’ transfers from another university or college to the nursing program affect their perceptions of cognitive processing approaches.

**Table 6:** Association of the socio-demographic characteristics of the students and Student perceptions about Cognitive processing strategies Scale

socio-demographic characteristics	IAU University Score of motivation	Alexandria University score of motivation
	Mean ± SD	Mean ± SD
Gender		
Male	86.4 ± 27.2	94.6 ± 24.0
Female	94.6 ± 19.8	93.9 ± 17.1
T [p]	1.468 [0.146]	0.129 [0.898]
Marital Status		
Not Married	92.7 ± 22.9	94.2 ± 19.0
Married	88.6 ± 20.2	92.6 ± 13.4
T [p]	0.554 [0.581]	0.262 [0.794]
Age (Years)		
20 < 21	83.3 ± 22.4	100.8 ± 14.5
21 < 22	95.6 ± 18.4	98.1 ± 17.6
22 < 23	89.6 ± 35.3	97.6 ± 19.5
> 23	114.3 ± 15.9	90.5 ± 18.7
F [p]	2.655 [0.055]	1.736 [0.164]
GPA		
< 3.6	86.4 ± 20.7	92.9 ± 18.7
3.6–4.5	92.6 ± 23.0	102.2 ± 15.6
> 4.5	92.4 ± 21.9	102.0 ± 17.0
F [p]	0.170 [0.844]	1.427 [0.245]
On average, how many hours per day do you spend studying (Hours)		
≤ 1	95.5 ± 31.6	87.2 ± 19.8
3–4	92.6 ± 23.0	95.3 ± 19.1
5–6	92.1 ± 15.7	96.9 ± 17.3
≥ 7	86.1 ± 20.5	88.5 ± 11.2
F [p]	0.292 [0.831]	1.215 [0.308]
Did you transfer from another university or college into the nursing?		
No	90.2 ± 22.1	95.4 ± 17.1
Yes	101.1 ± 23.1	83.0 ± 26.6
T [p]	1.596 [0.115]	2.130 [0.036]

IAU: Imam Abdulrahman bin Faisal University one way ANOVA test p value < 0.001

## Discussion

A nurse’s competency involves various components, such as academic concepts, professional judgment, a combination of skills, moral and ethical values, and the synthesis of all elements to define holism [23]. Educating students on thinking critically has become a crucial issue in today’s environment and has been represented as one of the core educational goals. Competence-based learning allows the incorporation of functionalist, behaviorist, and constructivist learning theories. Constructivist curricula attempt to allow students to learn new knowledge using their mental processes and in light of their previous learning experiences. As a result, students are expected to acquire questioning and thinking skills while learning [58]. Coronavirus (COVID-19) has caused chaos in educational systems worldwide. It resulted in a succession of pedagogical reforms that presented both opportunities and risks to educational quality [59]. So the present study aimed to assess students’ perceptions about critical thinking as well as the impact on motivation and learning strategies in the online psychiatric and mental health nursing program among Egyptian and Saudi undergraduates.

It can be noticed that both Egyptian and Saudi undergraduates displayed high mean scores in different subdomains of critical thinking motivation. The evidence suggests that students’ perceptions of critical thinking motivation were similar in the two cultures. It includes the expectations that all participants have about critical thinking (expectancy), the value of this skill (attainment), the benefit of using that skill (utility), how much I care and love to use that skill (interest), and their willingness to put time and effort into using that skill (cost domain). Furthermore, comparing both studied subjects revealed no statistically significant difference between them. This may be because teaching a psychiatric and mental health nursing course heavily depends on simulating reality by using different vignettes and case studies and urging students to discuss and interact by reflecting on their learning experiences and giving narrative feedback. Thus, this increased the students’ level of critical thinking, even if teaching was done online [60].

The same was reported by Guise et al., (2012), who indicated that the simulation type is well-suited to fostering critical thinking, communication, and decision-making skills in mental health nurses. It is also said that it is well-suited to e-learning and mixed-learning environments and is an excellent tool for multilingual simulations [61]. Similarly, it was found in different studies [62, 63]. The expansion of online courses in universities, partly due to the COVID-19 epidemic, has created a new environment for learning, allowing students to complete course-related work at their leisure and in their preferred location. This new setting may be beneficial or troublesome, depending on students’ motivation and ability to self-regulate their learning [64]. With the current epidemic states of COVID-19 raising the need for online psychiatric and mental health nursing, IAU college professors have been charged with developing dynamic and contemporary online clinical curricula by applying critical thinking and solving clinical problems. The course coordinators have worked on increasing the interaction and engagement of students, even if it was online, by making a link between the goals and future expectations. The anticipation and management of emotions are considered critical elements of success in an educational system, and teachers emphasize the importance of adopting a new mode in an educational system. This may also be due to the opportunities given to students to construct different alternatives to stimulate the natural environment.

There is a strong relationship between research competencies, age, and nursing research course grades. Grande et al., 2021, used a CBE approach to investigate the predictors and associations of Saudi nursing students’ research competencies. The majority of nursing students were already familiar with the nursing research process. Nursing research competency was significantly predicted by age and nursing research course grades (A+/A). As a result, students with A + or A grades were three times more likely to be competent than those with lower grades. Similarly, nursing students over 20 are twice as likely as those under 20 to be competent in research [23].

The present study also revealed that students displayed a high mean score in different cognitive processing strategies, including when I study for this class, I repeat the materials to myself several times. I make lists of essential items for this class and memorize the lists. When I study for this class, I gather information from different sources, such as lectures, readings, and discussions. I relate the materials to what I already know when studying for this class. When I study for this class, I write brief summaries of the main ideas from the materials and my class notes. I try to understand this class’s materials by connecting the readings and the concepts from the lectures. I apply ideas from other class activities, such as lectures and discussions. I make simple charts, diagrams, or tables to help me organize class materials. When I study for this class, I review my class notes and outline essential concepts. I often question things I hear or read in this class to decide if I find them convincing.

Furthermore, they reflect the use of cognitive processing strategies concerning a theory or conclusion when presented in class or in the readings, making decisions with supporting evidence, developing their own ideas, and so on. Fajari, (2020) studied students’ critical thinking abilities and the impact of learning motivation on critical thinking skills [65]. The findings of this study revealed that learning motivation has an impact on critical thinking abilities. Students’ critical thinking skills improve as their motivation to learn increases.

The modes of learning and instructing have a direct impact on each other. Three distinct learning styles affect learners’ cognitive information in eight aspects: the alignment of learners’ liberal and conservative values. Loyens, Rikers, & Schmidt, (2008) examined the relationship between students’ conceptions of constructivist learning and their regulation and processing strategies. They examined the relationship between students’ perceptions of constructivist learning and their regulation and processing strategies [66]. The findings revealed that constructivist learning and regulations, as well as processing techniques, have structural relationships. Furthermore, students who are unsure of their learning abilities are more likely to select an ineffective regulating strategy.

Generally, the present study finding may be explained by several aspects, including the students’ accustomedness to this system in teaching since the occurrence of the Corona pandemic, or it may be attributed to the efficiency of the performance of faculty members in the online teaching classroom management, having gone through the actual experience. This is in addition to the work of universities to develop the capabilities of faculty, staff, and students in managing the new requirement, not only because of the pandemic but also because this is in line with the needs of the new labor market requirement. In closing, combining online learning with a motivational approach to students will encourage participants to actively engage in critical thinking and cognitive processing processes.

## Conclusion

This study found that using a motivating method for online learning inspired students to engage in critical thinking and cognitive processing strategies in a psychiatric and mental health nursing course, even in two different contexts.

### Limitations of the study

The sample of this study was limited in number because it was obtained from nursing students in specific nursing colleges at Imam Abdulrahman Bin Faisal University in Saudi Arabia and Alexandria University in Egypt who undertook a specific psychiatric and mental health nursing course. The results obtained from this study may not apply to students studying different online nursing courses. Furthermore, this quantitative study does not help us better understand students` perceptions of critical thinking, motivation, and learning strategies in online psychiatric and mental health nursing education. In the future, it can be further improved by adding qualitative research.

### Recommendation

The recommendations for future study should broaden the subject by including more areas, more significant samples, and additional baccalaureate nursing programs. In addition, more investigation is required into how online learning experiences are perceived by both learners and educators and how this impacts how higher-level reasoning is developed in psychiatric nursing students. Several evaluations throughout the four-year nursing curriculum and the retention of critical thinking data by individual students would enable the monitoring of longitudinal developmental trends, individually and collectively, by class, gender, or any other crucial student factors.

### Implication in nursing practices

This research was one of the few distinctive areas of psychiatric nursing education. Because there is a lack of broad agreement about the progress and assessment of students’ perceptions of critical thinking and motivation learning strategies in this course because there are no evidence-based best practices for online learning, conventional ways of teaching psychiatric nursing have persisted, possibly failing to meet the demands of today’s technologically aware psychiatric nursing students in the future. Additionally, there is a need for a more recent study on online learning’s efficacy regarding the growth of critical thinking abilities and motivation through the use of online modalities in different nursing courses.

## Electronic supplementary material

Below is the link to the electronic supplementary material.

Supplementary Material 1

## Data Availability

The datasets used and/or analyzed during the current study available from the corresponding author on reasonable request.
