# Supplementary material for: Assessing perceptions about critical thinking, motivation learning strategies in online psychiatric and mental health nursing education among Egyptian and Saudi undergraduate nursing students
Source: BMC Nurs. 2023 Apr 10;22:112. doi: 10.1186/s12912-023-01264-2 (PMC10084656; doi:10.1186/s12912-023-01264-2)
Supplement: Supplementary file 1 — Supplementary Material 1 [file 12912_2023_1264_MOESM1_ESM.docx]

‘Student perceptions about critical thinking motivation and learning strategies in online psychiatric and mental health nursing education

You are invited to participate in a web-based online survey on *‘*Students’ perceptions about critical thinking in online nursing education: A comparative study.". This is a research project being conducted by **Mrs. Nora Al-Otaibi, Dr. Eman Sameh, Dr. Neama Kamel , Dr. Amira al-Showkan and Dr. Ayman Mohamed El-Ashry**, at College of Nursing in IAU.

It should take approximately less than 30 [minutes] to complete and consist of 2 parts including**:**

Sociodemographic data,

**First:** Critical Thinking Motivational Scale (19 statements)

**Second:** Cognitive processing Scale (18 statements).

**PARTICIPATION**
Your participation in this survey is voluntary. You may refuse to take part in the research or exit the survey at any time without penalty. You are free to decline to answer any particular question you do not wish to answer for any reason.

**BENEFITS**
You will receive no direct benefits from participating in this research study. However, your responses may help us learn more about your experience about critical thinking motivation and learning strategies in online nursing education this year.

**RISKS**

There are no foreseeable risks involved in participating in this study.
**CONFIDENTIALITY**
Your survey answers will be sent to a link at docs.google.com/forms/ where data will be stored in a password protected electronic format. A Google form does not collect identifying information such as your name, email address, or IP address. Therefore, your responses will remain anonymous. No one will be able to identify you or your answers, and no one will know whether or not you participated in the study.

**CONTACT**
If you have questions at any time about the study or the procedures, you may contact the primary investigator, Mrs. Nora Al-Otaibi via email at [ngalotaibi@iau.edu.sa].

If you have any queries or you would like to know your rights as a participant, you can contact the institutional review board via the following email: [irb@iau.edu.sa](mailto:irb@iau.edu.sa)

**ELECTRONIC CONSENT**: Please select your choice below. You may print a copy of this consent form for your records. Clicking on the “Agree” button indicates that

- You have read the above information
- You voluntarily agree to participate

**Socio-Demographic Questionnaire**

Directions: Please select each answer that applies to your situation.

**1. Gender**

Female

Male

**2. Present marital status**

Never Married

Married

Divorced

Widowed

**3. Age.**

**4. Current GPA**

2 or below

1-3.5

6 – 4.5

More than 4.5

**5. On average, how many hours per day do you spend studying?**

hour or less

3-4 hours

5-6 hours

7 or more hours

**7. Did you transfer from another university or college into the nursing?**

NO

Yes

**if yes what level?**

**Critical Thinking Motivational Scale**

The scale was developed that measures the different components of motivation with respect to critical thinking. This scale includes 19 Likert-type items with scores from 1 to 6, concerning which the subjects are requested to express their degree of agreement or disagreement with a series of statements.

**1. Concerning reasoning correctly, I am better than most of my peers.**

1 Strongly disagree

2

3

4

5

6 Strongly agree

**2. I feel capable of understanding everything related to thinking in a rigorous way.**

1 Strongly disagree

2

3

4

5

6 Strongly agree

**3. I am able to learn how to think in a rigorous way.**

1 Strongly disagree

2

3

4

5

6 Strongly agree

**4. I am able to learn how to reason correctly better than most of my peers.**

1 Strongly disagree

2

3

4

5

6 Strongly agree

**5. For me it is important to learn how to reason correctly.**

1 Strongly disagree

2

3

4

5

6 Strongly agree

**6. For me it is important to be good at reasoning.**

1 Strongly disagree

2

3

4

5

6 Strongly agree

**7. For me it is important to use my intellectual skills correctly.**

1 Strongly disagree

2

3

4

5

6 Strongly agree

**8. For me it is important to be good at solving problems.**

1 Strongly disagree

2

3

4

5

6 Strongly agree

**9. Thinking critically will help me to become a good professional.**

1 Strongly disagree

2

3

4

5

6 Strongly agree

**10. Thinking critically will be useful for my future.**

1 Strongly disagree

2

3

4

5

6 Strongly agree

**11. Thinking critically is useful in everyday life.**

1 Strongly disagree

2

3

4

5

6 Strongly agree

**12. Thinking critically is useful for other subjects and courses.**

1 Strongly disagree

1

2

3

4

5

6 Strongly agree

**13. I like to reason properly before deciding about something.**

1 Strongly disagree

2

3

4

5

6Strongly agree

**14. I like to learn things that will improve my way of thinking.**

1 Strongly disagree

2

3

4

5

6 Strongly agree

**15. I like thinking critically. I like to reason in a rigorous manner.**

1 Strongly disagree

2

3

4

5

6 Strongly agree

**16. Thinking critically will help me to become a good professional.**

1 Strongly disagree

2

3

4

5

6 Strongly agree

**17. If I have a problem that requires me to reason in a critical way, I am disposed to sacrifice the time that I would otherwise have devoted to other things.**

1 Strongly disagree

2

3

4

5

6 Strongly agree

**18. I am disposed to sacrifice quite a lot of time and effort in order to improve my way of reasoning.**

1 Strongly disagree

2

3

4

5

6 Strongly agree

**19. It is worth investing time and efforts to acquire and use critical thinking.**

1 Strongly disagree

2

3

4

5

6 Strongly agree

**Cognitive processing strategies Scale**

The cognitive processing strategies scale composed of 18 itemsو All the items were on a seven-point Likert scale, with one representing “not at all true of me” and seven “very true of me.” , concerning which the subjects are requested to express their degree of satisfied or disagreement with a series of statements

**1. When I study for this class, I practice saying the materials to myself over and over.**

1 Not at all true of me

2

3

4

5

6

7 Very true of me

**2. I make lists of important items for this this class and memorize the lists.**

1 Not at all true of me

2

3

4

5

6

7 Very true of me

**3. When I study for this class, I pull together information from different sources such as lectures, readings, and discussions.**

1 Not at all true of me

2

3

4

5

6

7 Very true of me

**4. When studying for this class, I try to relate the materials to what I already know.**

1 Not at all true of me

2

3

4

5

6

7 Very true of me

**5. When I study for this class, I write brief summaries of the main ideas from the materials and my class notes.**

1 Not at all true of me

2

3

4

5

6

7 Very true of me

**6. I try to understand the materials in this class by making connections between the readings and the concepts from the lectures.**

1 Not at all true of me

2

3

4

5

6

7Very true of me

**7. I try to apply ideas from other class activities such as lectures and discussions.**

1 Not at all true of me

2

3

4

5

6

7 Very true of me

**8. I make simple charts, diagrams, or tables to help me organize class materials.**

1 Not at all true of me

2

3

4

5

6

7Very true of me

**9. When I study for this class, I go over my class notes and make an outline of important concepts.**

1 Not at all true of me

2

3

4

5

6

7 Very true of me

**10. I often find myself questioning things I hear or read in this class to decide if I find them convincing.**

1 Not at all true of me

2

3

4

5

6

7 Very true of me

**11. When a theory, interpretation, or conclusion is presented in class or in the readings, I try to decide if there is good supporting evidence.**

1 Not at all true of me

2

3

4

5

6

7 Very true of me

**12. I treat the class materials as a starting point and try to develop my own ideas about it.**

1 Not at all true of me

2

3

4

5

6

7 Very true of me

**13. Whenever I read or hear an assertion or a conclusion in this class, I think about possible alternatives.**

1 Not at all true of me

2

3

4

5

6

7 Very true of me

**14. When studying for this class, I make up questions to help focus on learning materials.**

1 Not at all true of me

2

3

4

5

6

7 Very true of me

**15. When I become confused about something I’m studying for this class, I go back and try to figure it out.**

1 Not at all true of me

2

3

4

5

6

7 Very true of me

**16. If the class materials are difficult to understand, I change the way I study the materials.**

1 Not at all true of me

2

3

4

5

6

7 Very true of me

**17. When I study for this class, I set goals for myself in order to direct my activities in each study period.**

1 Not at all true of me

2

3

4

5

6

7 Very true of me

**18. If I get confused taking notes in class, I make sure I sort it out afterwards.**

1 Not at all true of me

2

3

4

5

6

7 Very true of me
